# Early Development of Spinal Deformities in Children Severely Affected with Spinal Muscular Atrophy after Gene Therapy with Onasemnogene Abeparvovec—Preliminary Results

**DOI:** 10.3390/children10060998

**Published:** 2023-06-02

**Authors:** Venla Soini, Gudrun Schreiber, Bernd Wilken, Anna Kathrin Hell

**Affiliations:** 1 Paediatric Orthopaedics, Department of Trauma, Orthopaedic and Plastic Surgery, University Medical Center Göttingen, 37075 Göttingen, Germany; veviso@utu.fi; 2 Department of Paediatric Surgery and Paediatric Orthopaedic Surgery, University of Turku and Turku University Hospital, FI-20520 Turku, Finland; 3 Department of Pediatric Neurology, Social Pediatric Center, Medical Center Kassel, 34127 Kassel, Germany; gudrun.schreiber@gnh.net (G.S.); bernd.wilken@gnh.net (B.W.)

**Keywords:** spinal muscular atrophy, scoliosis, kyphosis, gene therapy

## Abstract

Spinal muscular atrophy (SMA) is a rare genetic disorder, with the most common form being 5q SMA. Survival of children with severe SMA is poor, yet major advances have been made in recent years in pharmaceutical treatment, such as gene-therapy, which has improved patient survival. Therefore, clinical problems, such as the development of spinal deformities in these genetically treated SMA children represent an unknown challenge in clinical work. In a retrospective case series, the development of spinal deformities was analyzed in 16 SMA children (9 male, 7 female) treated with onasemnogene abeparvovec in two institutions during the years 2020 to 2022. Ten out of sixteen patients had a significant kyphosis, and nine out of sixteen patients had significant scoliosis, with the mean curvature angles of 24 ± 27° for scoliosis, and 69 ± 15° for kyphosis. Based on these preliminary data, it can be assumed that early-onset kyphosis presents a clinical challenge in gene-therapy-treated SMA children. Larger datasets with longer follow-up times need to be collected in order to verify these preliminary observations.

## 1. Introduction

Spinal muscular atrophy (SMA) is a group of hereditary neuromuscular diseases with a range of different genotypes. The most common is 5q SMA, which includes mutations in the *SMN1*-gene and covers approximately 95% of the spectrum of SMA cases [1,2]. It leads to progressive muscle weakness and atrophy due to loss of lower motor neurons [3]. SMA forms caused by mutations in other genes are called non-5q-SMAs [4]. The severity of the disorder manifests from mild (SMA type 4) to severe (SMA type 1), and the clinical severity depends on the number of copies of the similar but less-stable *SMN2*-gene [5,6,7]. Previously, scoliosis has possessed a major problem to SMA type 2 patients [8,9], and the poorer status of ambulation correlates to more severe deformity [10]. The prognosis of SMA type 1 newborns was poor, with early death in most cases before the emergence of drug treatment options. Therefore, spinal deformity was generally neither seen nor treated in this patient group [11,12].

Novel drug options such as the antisense oligonucleotide nusinersen or survival motor neuron splicing modifying risdiplam have provided a significant improvement to patient prognosis. Nonetheless severe scoliosis may occur in patients with SMA types 1 and 2 at a young age [8,10]. Previous literature states that the novel drug therapies have shifted the focus of scoliosis incidence from SMA type 2 patients more towards to SMA type 1 children [13,14].

In addition to the pharmaceutical therapy, the comprehensive treatment plans include bracing, physiotherapy, and surgery, such as growth-friendly spinal implants, and eventually spinal fusion in adolescents [15].

One of the latest advances in drug therapy of SMA is gene therapy, the effects of which are delivered through an adeno-associated viral serotype 9 (AAV9) vector [16,17]. Onasemnogene abeparvovec–xioi (Zolgensma^®^) was approved in Europe by the EMA on 18 May 2020 [18]. The therapy requires a one-time injection, after which an AAV9 vector delivers the *SMN1*-gene through the blood–brain barrier to motor neuron cells into the central nervous system. The indications are limited due to the massive costs [19,20,21,22,23]. Currently, there are two indications for receiving the drug: 5q SMA due to biallelic *SMN1*-gene mutation and clinical diagnosis of SMA type 1, or 5q SMA due to biallelic *SMN1*-gene mutation and a maximum of four copies of *SMN2*-genes in patients less than two years of age.

After approval, the drug has been adapted in clinical practice, and the patient results have been promising; improvements in the ability of sitting and walking and ventilation in terms of weaning from noninvasive ventilation have been previously reported [24,25,26,27,28]. However, little is known about the effects of onasemnogene abeparvovec on the natural history of neuromuscular spinal deformities in SMA children. Early-onset scoliosis has been observed in gene-therapy-treated SMA individuals [29]. The aim of this investigation was to examine the development of early-onset kyphosis and scoliosis in SMA children treated with onasemnogene abeparvovec.

## 2. Materials and Methods

A case series of 16 SMA children treated with onasemnogene abeparvovec is presented. The Institutional Ethics Committee of University Medical Center Göttingen approved the study on 17 August 2015 (reference number DOK_246_2015). A retrospective review of the treated patients was performed to find out whether spinal deformity poses a problem in this young SMA patient population. This cohort was enrolled for treatment of SMA in two different tertiary level institutes after the launching of onasemnogene abeparvovec in 2020. In center 1, we retrieved patient information from the database based on diagnosis codes, G12.0 (Infantile spinal muscular atrophy, type I Werdnig–Hoffman), G12.1 (Other inherited spinal muscular atrophy), G12.2 (Spinal muscular atrophy and related syndromes), G12.8 (Other spinal muscular atrophies and related syndromes), G12.9 (Spinal muscular atrophy, unspecified), and identified 118 pediatric patients with the diagnoses in question. We screened the medical records of these patients and excluded 23 for non-SMA muscle disease diagnosis. Of the remaining 95 patients, those 20 born after 2017 were included for a more detailed review, as children born before 2017 have not had access to gene therapy in Germany. After the review of all medical records in question, 11 SMA children treated with onasemnogene abeparvovec were included in the analysis. To receive more volume to our cohort, we collaborated with another clinic in the area, and screened their institutional registry, through which eight more patients were collected for further analysis. Three patients were excluded from the curvature analysis due to lack of spinal radiographic imaging, leading to a total of 16 patients in this case series.

We collected data concerning patient demographics, SMA characteristics, and spinal deformity development from the patients’ birth until present records. Patients were divided into scoliosis and non-scoliosis groups based on the diagnostic threshold limit of 10° scoliosis angle. Furthermore, a distinction between clinical kyphosis and non-kyphosis was made based on the data of age-dependent kyphosis presented in the publication of Giglio and Volpon, 2007 [30]. Age-adjusted hyper-kyphosis was calculated by subtracting the age-dependent normal curve (25° + 0.58 × [age in years]) from the measured kyphosis angle. Based on calculated age-adjusted hyper-kyphosis angle, we divided the patients into kyphosis and no-kyphosis subgroups, with a threshold limit of 10°. 

Statistical analysis was performed to observe the differences between patients. All analysis was performed with JMP Pro 16.2. Distribution and bivariate analysis were carried out in order to examine the correlations of gene therapy and spinal deformities. One-way ANOVA was performed for categorical data, and the Wilcoxon Rank sum test was used in cases with non-parametric data. Linear fit was applied for continuous data. The statistical significance threshold was set at <0.05. 

## 3. Results

Out of the 16 patients included in our dataset, 9 were male and 7 were female. Twelve (75%) of the patients were diagnosed with SMA type 1, and four (25%) patients with SMA type 2. There were no SMA type 3 or SMA type 4 patients included in this study. All patients had a homozygous deletion of the *SMN1*-gene. The number of *SMN2*-gene copies were two copies for 11 patients (69%), and three copies for 5 patients (31%). The mean age for receiving onasemnogene abeparvovec was 1.54 ± 1.34 years. A total of nine patients (56%) had received pharmaceutical therapy with nusinersen before onset of treatment with onasemnogene abeparvovec, and two patients (13%) had received risdiplam prior to onasemnogene abeparvovec. None of the patients had received both nusinersen and risdiplam prior to gene therapy (Table 1).

The current clinical situation was recorded at the timepoint of the last available spine radiographs in the database. The mean age at the last record point was 3.0 ± 1.6 years and the time from the onset of gene therapy to the last imaging was 1.5 ± 0.8 years. 

Fourteen (88%) of the patients were non-ambulatory, one child (6%) was ambulatory, and one child was using aids for walking. Three patients (19%) used non-invasive ventilation at night-time, and one patient (6%) needed continuous ventilation. At the time of the last follow-up with radiographs, the mean height was 92 ± 11 cm, and the mean weight 13.3 ± 3.4 kg.

Spinal deformity was analyzed on standardized anterior–posterior (ap) and lateral radiographs in a sitting position. Due to radiation protection in these young children, radiographs were only taken if spinal deformities were visible clinically. All 16 SMA children received an ap radiographic exam, while lateral radiographs were only taken in ten (63%) SMA children. 

Even though scoliosis seemed to be present in all SMA children, seven (44%) patients did not have a notable scoliosis on ap radiographs. The remaining nine children (56%) presented with a mean scoliosis angle of 24 ± 27°. Six patients (38%) had a large, C-shaped curve, while three SMA children (19%) presented with an S-shaped scoliosis.

**Figure 1:**
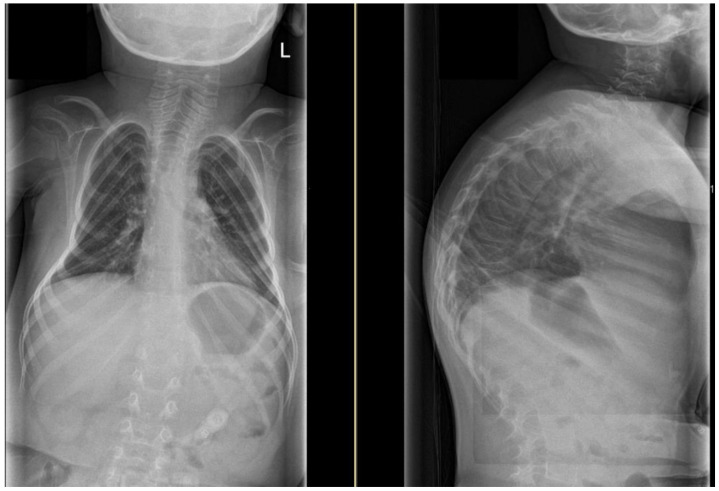
Anteroposterior and lateral sitting radiographs of patient no. 13.

All patients with lateral radiographic imaging (n = 10; 63%) had a significant kyphosis (Figure 1). The deformity angles of patients with kyphosis and/or scoliosis are listed in Table 2. The mean scoliosis angle was 24 ± 27°, the kyphosis angle was 69 ± 15°, and the age-adjusted hyper-kyphosis angle was 42 ± 16° above normal age-related kyphosis values (Table 2).

There was a positive correlation between the onset of onasemnogene abeparvovec treatment to the latest scoliosis curvature angle (correlation coefficient = 0.432), yet this was not statistically significant (*p* = 0.06). The mean age of receiving onasemnogene abeparvovec was higher in patients with scoliosis in comparison to patients without scoliosis (mean age 1.98 ± 0.40 vs. 0.99 ± 0.49, respectively), yet this finding was not statistically significant (*p* = 0.489). The same phenomenon was visible for kyphosis, with onasemnogene abeparvovec treatment-onset age means of 1.91 ± 0.38 for kyphotic and 0.93 ± 0.53 for non-kyphotic patients. Again, there was no statistical significance (*p* = 0.173). The follow-up time between gene therapy and the analyzed radiographs did not correlate with kyphotic or scoliotic angles.

*SMN2*-gene counts did not show an influence on scoliotic angles. Patients with two *SMN2*-genes showed larger kyphotic angles in comparison to patients with three *SMN2*-genes (mean kyphosis angle 77 ± 5° vs. 57 ± 6°, *p* = 0.033). The same finding was also seen in age-adjusted kyphosis angles, with mean values of 51 ± 5 ° for children with two *SMN2*-gene counts and 29 ± 6° for patients with three *SMN2*-gene counts (*p* = 0.043).

SMA subtypes, weight, height, ventilation status, or sex did not significantly influence the development of spinal deformities. Additionally, neither the type or onset of earlier pharmaceutical therapy nor the current age showed any significant impact on the existence and/or degree of scoliosis or kyphosis. All scoliotic patients were non-ambulatory, but this could not be verified statistically. The emergence of scoliosis and kyphosis did not correlate to each other in a statistically significant way.

## 4. Discussion

To the best of our knowledge, this is the first paper to describe the natural history of kyphotic spinal deformities in young SMA children after onasemnogene abeparvovec therapy, and also adds to the literature concerning scoliosis in gene-therapytreated SMA children.

The main limitation of this cohort is the small number of patients due to the rarity of the disease, which leads to the descriptive nature of this study. Another notable limitation is the fact that onasemnogene abeparvovec treatment has only been available for a short time period, so our follow up time is also rather short. There was a great variation from the time of gene therapy to the radiographic imaging. Furthermore, upright lateral radiographs were not available from all of the patients. Additionally, our center is specialized in pediatric spinal deformities, and we recognize that this might affect the patient selection, as all the patients with milder spinal deformities might not appear in our center. However, the use of the patient register of another non-specialized institute reduces the impact of this bias. In addition, the initial search of patients included all available patients with SMA-related diagnosis, as listed in method section, regardless of the occurrence of spinal deformities. 

However, in our opinion, the phenomenon of early-onset spinal deformity in SMA patients who previously received gene therapy is important and deserves to be highlighted based on preliminary trends.

A notable proportion of 63% (n = 10) of the gene-therapy-treated SMA patients developed severe thoracic kyphosis early in life. The natural development of kyphosis in SMA children has been acknowledged in the literature, and Riddick et al. stated in 1982 that kyphosis may develop before the age of three years with clinically significant scoliosis or kyphoscoliosis thereafter [31,32]. The new treatment regime with onasemnogene abeparvovec causes the survival of previously fatally affected SMA type 1 children. As a consequence, this patient population will be mobilized early in life with a relatively large head and a hypotonic trunk. Again, this will lead to hyperlordosis of the cervical spine and hyperkyphosis of the thoracic spine. Reduced mineral bone density, which is caused by the disease itself as well as due to immobilization [33,34], will add to this problem. Morphometric changes of the normal spinal anatomy and spinal deformities such as thoracic hyperkyphosis will diminish the height of the thorax, which will lead to thoracic insufficiency syndrome due to lung function and volume reduction [35].

The mean age for receiving gene therapy of the patients who developed a scoliosis was higher (mean 1.98) than those who had not developed scoliosis yet (mean 0.99). This is in line with the observation that early treatment will lessen the severity of the disease. Previous studies have stated that the pharmaceutical treatments for SMA are most effective if administered immediately after or even before the onset of clinical symptoms [14,36]. Therefore, a newborn screening has been proposed to be the most effective way to ensure early diagnosis [37]. Concerning newborn screening in practice, the European Alliance of Newborn Screening in Spinal Muscular Atrophy has demanded a mandatory routine newborn screening of SMA as part of clinical practice by the year 2025 [38]. An SMA screening would allow onasemnogene abeparvovec treatment as early as possible. This would also possibly minimize the degree of spinal deformity development further on. 

Scoliosis is a well-recognized and researched clinical problem in SMA patients and is directly related to the disease severity [39,40,41], which makes it only logical that the drugs effecting the course of the disease have a beneficial influence on scoliosis and kyphosis. However, there is currently only limited literature analyzing this hypothesis. Stettner et al. recently published an observational study of nine pediatric SMA individuals treated with onasemnogene abeparvovec and found that 67% of their patients developed scoliosis in their follow up after approximately one year. Scoliosis angles ranged from 20 to 54°. They observed no association between the nutritional status or respiratory support and the development of scoliosis. They also stated that development of scoliosis must be thoroughly evaluated in SMA type 1 patients [29]. Our results are in line with these findings, as scoliosis was likewise evident in our cohort, and curve degrees were similar. 

The development of spinal deformities after application of other pharmaceutical therapies available for SMA has been studied. Al-Armani et al. demonstrated in their descriptive investigation that treatment with nusinersen showed initial improvements in functional parameters as measured by the CHOP-INTEND score (The Children’s Hospital of Philadelphia Infant Test of Neuromuscular Disorders), yet all of their patients still developed scoliosis during the first year of life [13]. Kotulska et al. speculated about the same issue in their review on SMA. Although overall motor development and survival of the pharmaceutically treated SMA patients improved, the patients still developed early-onset scoliosis [14]. In this study, the amount of fewer gene copies showed a statistical significant correlation with higher kyphotic curve angles. This is in line with the existing literature, as the amount of gene copies correlated with the disease severity [7,42]. However, it must be noted that the age of receiving onasemnogene abeparvovec was also clearly yet not statistically significantly lower in these patients with two *SMN2*-gene copies in comparison to three *SMN2*-gene copies (1.27 years vs. 2.1 years, *p* = 0.0529). This might have an impact on the incidence and severity of spinal deformities, and this should definitely be analyzed with larger datasets.

Current European guidelines recommend that onasemnogene abeparvovec gene therapy should ideally be given to children with SMA younger than six months and weighing up to 13.5 kg [36]. In this case series, patients were significantly older than this recommendation, yet the mean weight was in lines with the recommendation. In the future, spinal deformity progression needs to be further assessed in a cohort of children who received the treatment according to the current recommendations. 

Immediate administration-related adverse effects of onasemnogene abeparvovec include pyrexia and vomiting [43], acute liver failure [44], transient thrombocytopenia, and elevated troponin levels [14,45]. Additionally, fatal thrombotic microangiopathy has been described [46]. Long-term effects of the gene therapy are not clearly understood due to the short follow-up period available, and they might be hard to distinguish from the disease progression.

In kindergarten or primary school SMA children with progressive spinal deformity, growth-friendly spinal implants (GFSI) are currently used as an interim treatment option until definite spinal fusion can be performed beyond the age of ten years. There are several rib, spine, or pelvic fixation techniques on the market using repetitive surgery or external remote control to lengthen these implants over time [47,48,49,50]. However, several complications such as exposure to repetitive surgeries and risk of infection, autofusion and deformity stiffness are associated with most techniques. Some authors suggest early definite spinal fusion, while neglecting the negative effects on lung function and volume and thoracic growth [51].

In the described pediatric SMA population with small numbers of *SMN2*-gene copies and onasemnogene abeparvovec treatment, we believe that the majority of children will develop progressive spinal deformity in early childhood and will most likely need early GFSI treatment for deformity control. Kyphosis especially will not respond well to brace therapy, and braces may have additional adverse effects on lung function. As of now, three of the described patients (no. 7, 14 and 15) have undergone GFSI surgery with repetitive lengthening procedures, while the rest of this population will be monitored closely. However, poor bone stock, severely impaired head control, and low weight are limitations for surgical interventions. Therefore, treatment of these children with severe SMA remains a challenge despite overall better functional outcome and survival.

## 5. Conclusions

More patient observations and data, and longer follow-up times are needed to determine the effect of gene therapy with onasemnogene abeparvovec on the natural history of spinal deformities in pediatric patients with SMA. Based on our preliminary results, we hypothesize that severely affected SMA children—who formerly might not have survived—and who are now treated with onasemnogene abeparvovec develop early spinal deformity, especially hyperkyphosis. We suggest taking these findings into account when treating this patient population and underline the importance of a comprehensive clinical examination and care as well as taking sitting radiographs in two planes for detection of spinal deformity. 

## Figures and Tables

**Table 1:** Patient demographics of SMA children (n = 16) treated with onasemnogene abeparvovec.

ID	Sex	Earlier Treatment (Age in Years), Type of Treatment	Onasemnogene Abeparvovec Age (Years)	SMA Type	*SMN2* Gene Copies	Ambulation Status at the Time of Imaging	Age at Imaging (Years)
1	F	0.2, Nusinersen	0.2	1	2	non-ambulatory	1.6
2	F	0.1, Nusinersen	0.2	1	2	non-ambulatory	1.4
3	M	0.2, Risdiplam	0.3	1	2	non-ambulatory	1.1
4	F		0.3	1	2	non-ambulatory	1.5
5	F		0.7	1	2	non-ambulatory	1.6
6	F	0.5, Nusinersen	0.9	1	2	non-ambulatory	2.2
7	M	0.5, Nusinersem	1.3	1	2	non-ambulatory	2.3
8	M	0.4, Nusinersen	1.3	1	2	non-ambulatory	3.7
9	M	0.3, Nusinersen	1.4	1	2	non-ambulatory	3.5
10	M		1.5	2	3	non-ambulatory	3.6
11	M	1.3, Nusinersen	1.5	2	3	minimal ambulatory	4.0
12	M	-, Risdiplam	1.5	1	3	ambulatory	1.6
13	M	0.2, Nusinersen	1.7	1	2	non-ambulatory	5.6
14	M	1.6, Nusinersen	3.3	2	3	non-ambulatory	4.8
15	F		3.7	1	2	non-ambulatory	4.3
16	F		4.9	2	3	non-ambulatory	6.9

**Table 2:** Characteristics of spinal deformities in young SMA children treated with onasemnogene abeparvovec.

ID	Scoliosis Type	Scoliosis Curvature Angle (°)	Kyphosis Curvature Angle (°)
1	S-type	29	
2	S-type	43	66
3	no	7	86
4	no	6	
5	no	0	70
6	no	0	
7	left convex C-type	69	75
8	left convex C-type	24	
9	left convex C-type	52	60
10	left convex C-type	21	
11	no	5	
12	no	7	48
13	no	7	94
14	left convex C-type	64	73
15	right convex C-type	82	76
16	S-type	35	45

## Data Availability

Please contact author for data requests.
